# Epidemiology and Recurrence of Sigmoid Volvulus: Analysis of Health Insurance Claims Data in Japan

**DOI:** 10.1002/deo2.70212

**Published:** 2025-10-14

**Authors:** Masaaki Yamada, Michikazu Sekine, Haruka Fujinami, Yuchi Motofuji, Eiji Shinno

**Affiliations:** ^1^ Department of Epidemiology and Health Policy School of Medicine University of Toyama Toyama Japan; ^2^ Department of Gastroenterology Shinseikai Toyama Hospital Imizu Japan; ^3^ Department of Endoscopy Toyama University Hospital Toyama Japan

**Keywords:** endoscopic reduction, epidemiology, incidence, recurrence rate, sigmoid volvulus

## Abstract

**Objectives:**

Sigmoid volvulus (SV) is rare in Japan, and most studies have been limited to small numbers or case series reports. We aimed to explore the incidence, risk factors, and recurrence rates of SV in Japan using community‐based health insurance claims data.

**Methods:**

We used health insurance claims data from the Toyama Prefecture from April 2018 to March 2022. Individuals aged ≥ 40 years who had no diagnosis of SV (International Statistical Classification of Diseases, 10th Revision) in 2018 were included in our study. Patients who were newly diagnosed with SV in 2019 were identified. The annual incidence, risk factors, and recurrence rates of SV within 2 years of the first endoscopic reduction were evaluated. Poisson regression analysis was employed to identify risk factors, and Kaplan–Meier analysis was performed to analyze recurrence.

**Results:**

Of the 327,693 insured individuals in Toyama, 52 developed SV in 2019 (0.0159% = 15.9/100,000). Men (adjusted rate ratio [aRR] = 3.46, 95% confidence interval [CI]: 1.92– 6.25), constipation (aRR = 4.63, 95% CI: 2.50–8.58), and higher disability levels (level 2–3: aRR = 6.54, 95% CI: 2.43–13.57, level 4–5: RR = 2.94, 95% CI: 1.10–7.84) were significantly associated with SV incidence. Of the 45 patients who underwent only endoscopic reduction, recurrence within 2 years was observed in 16 (cumulative recurrence rate, 35.6%).

**Conclusions:**

SV is common in community‐based studies. Men, constipation, and a high disability level were risk factors. The recurrence rate of SV after endoscopic reduction was approximately 35% within 2 years. Our insurance claims data offered valuable insights into SV epidemiology.

## Graphical abstract

• An annual incidence rate of sigmoid volvulus (SV) was 0.0159% (15.9/100,000).

• After endoscopic reduction, the cumulative recurrence rate within 2 years was 35.6%.

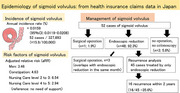

## 1 Introduction

Sigmoid volvulus (SV) refers to torsion of the bowel around its mesentery, leading to bowel obstruction, intestinal necrosis, or perforation if blood flow is obstructed [1]. The incidence varies by region. Among endemic regions with high incidence, such as Latin America, Africa, Eastern Europe, Russia, the Middle East, and India, that are labeled as “*volvulus belt*,” SV represents 20%–54% of all intestinal obstructions [2]. Conversely, SV in low‐incidence areas, such as North America, Western Europe, Australia, and Japan, accounts for only 3%–5% of all intestinal obstructions [2, 3]. However, most previous reports were based on hospital statistics rather than community‐based data. It is impossible to deduce the incidence of SV from hospital‐based data. To date, only one community‐based study in the USA between 1960 and 1980 has been conducted on SV, and the incidence was estimated to be 1.67 per 100,000 person‐years (8.76 in >60 years old) [4]. Given the drastic changes in the environment and population demography, updated surveys are necessary to determine the current incidence of SV.

In Western countries, such as the USA and Japan, risk factors for SV include older adults, men, diabetes, constipation, neuropsychiatric disease, and institutionalized patients [1, 2, 5]. These factors were considered to reduce bowel mobility, leading to the development of SV. Given that most studies used data from a single hospital or a few hospitals, the risk factors may differ from those used in community‐based studies. Endoscopic reduction is the first‐line therapy for uncomplicated SV because it is less invasive and allows clinicians to differentiate between viable and nonviable bowel by visualizing the sigmoid colon [1, 2, 6]. However, a limited number of studies have reported the recurrence rate of SV after the first endoscopic reduction, ranging from 36.1% to 61.4% [7, 8, 9]. Estimating the recurrence rate can be beneficial for gastrointestinal (GI) specialists in managing SV, particularly as the number of older patients unfit for surgical procedures continues to increase in developed countries.

Therefore, our study aimed to investigate (1) the incidence rate and (2) recurrence rate of SV after the first endoscopic reduction using community‐based health insurance claims data in Japan.

## 2 Methods

### 2.1 Data Source and Study Design

This cohort study used two health insurance claims datasets from April 2018 to March 2022: the National Health Insurance Claim, “*Kokuho*,” which mainly covers persons aged 60–74 years, and the Advanced Elderly Medical Service System, “*Kouki Koureisha Iryo Seido*,” which covers persons aged 75 years or older, both maintained by Toyama Prefecture in Japan. The two health insurance claims datasets contain monthly health insurance claims, including all procedural codes, International Statistical Classification of Diseases, 10^th^ Revision (ICD‐10) codes, and prescriptions of inpatients and outpatients [10]. The database contains an extremely large‐scale population, enabling the study of relatively rare disease entities, such as SV. We also used the Long‐Term Care Insurance “*Kaigo*” data of Toyama Prefecture, which contains monthly service benefits for care of older people and the disability level of each individual [11].

### 2.2 Study Populations

**FIGURE 1:**
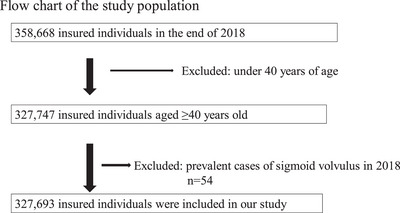
Flow chart of the study population. At the end of the 2018 fiscal year, out of the 358,668 individuals insured under “*Kokuho*” and “*Kouki Koureisha Iryo Seido*,” those aged ≥ 40 years were included. Patients who had already been diagnosed with SV in 2018 were excluded. Finally, 327,693 insured persons were analyzed in our study.

Toyama Prefecture is located in the central part of Japan (Chubu region), with about 1 million people as of 2019, of which approximately 32% were older adults (aged **≥**65 years) [12]. Our data included 358,668 persons insured by the “*Kokuho*” and “*Kouki Koureisha Iryo Seido*” at the end of the 2018 fiscal year. Because the mean age of SV in most studies is in the 60s or 70s and the condition is less common in younger individuals [5, 8], those younger than 40 years of age were excluded. Patients who had been diagnosed with SV in 2018 were excluded from this study. Finally, 327,693 insured individuals were included in this study. A flowchart of the process is shown in Figure 1.

### 2.3 Definition of Cases and Other Variables

SV cases were identified using the diagnostic codes of ICD‐10 (K562 and 20050371). Endoscopic reduction (J034‐03), colonoscopy (D313), and abdominal surgery (K714, 719, 726, and 735) were identified using the treatment (or billing) codes of Japan's Ministry of Health, Labor, and Welfare (MHLW) (Table S1). One patient was diagnosed with SV and underwent a colonoscopy within the same month. We defined the patient as having undergone endoscopic reduction because the billing code for endoscopic reduction (J034‐03) started in 2018, and was not familiar to some clerical staff.

Comorbidities, such as hyperlipidemia, hypertension, diabetes, psychiatric diseases, and constipation, were defined by the prescription of the corresponding drugs from the drug classification code of Japan's MHLW (218 for hyperlipidemia, 214 for hypertension, 396 for diabetes, and 117 for psychiatric diseases) (Table S1).

The disability level was diagnosed based on the classification of Long‐Term Care Level, which was certified by each municipality in Japan [11]. This system uses a 74‐item questionnaire assessing activities of daily living and categorizes individuals into seven levels of long‐term care certificates: Support levels (“*YouShienn*”) 1 and 2, and Nursing Care levels (“*YouKaigo*”) 1 to 5 [13]. Higher levels indicate greater disability severity. For example, Support level 2 or Nursing Care level 1 reflects a need for partial physical or cognitive support, such as assistance with shopping. Nursing Care level 3 reflects a need for help with activities such as changing clothes or using the restroom. In this study, the disability level was divided into 4: independent (no need for support), Support care to Nursing Care level 1, Nursing Care levels 2–3, and Nursing Care levels 4–5 (severely dependent).

### 2.4 Recurrence Analysis After the First Endoscopic Reduction

We followed the patients who only underwent endoscopic reduction for 2 years until March 2022, and investigated their recurrence period (months). Recurrence period of SV after the first endoscopic reduction was determined by the reappearance of the diagnostic code or the first appearance of the abdominal surgery treatment code from the person‐month claim data. Patients who had an SV diagnostic code in consecutive months were not defined as a recurrence because we could not discern between only one onset or a recurrence of SV.

### 2.5 Statistical Analysis

The participants were divided into four age groups (40–69, 70–79, 80–89, and 90–). The incidence rates of SV were expressed according to age, sex, comorbidities, and disability levels using the chi‐square test (Table 1). Variables with a *p‐*value <0.05 in the chi‐square test were inserted in a multivariable Poisson regression analysis as independent factors to clarify the risk factors of SV (Table 2). Adjusted risk ratios (aRRs) with 95% confidence intervals (CIs) were calculated. In the recurrence analysis, the cumulative recurrence rate of SV after the first endoscopic reduction, time to recurrence was plotted using the Kaplan–Meier method, and the relationship between the recurrence rate and basic characteristics (sex and age categories) was compared using the log‐rank test. Data were analyzed using IBM Statistics (ver.26; IBM Corp., USA) and STATA (ver. 16; STATA Corp., College Station, TX, USA). Statistical significance was set at *p* < 0.05.

**TABLE 1:** Characteristics and the incidence of sigmoid volvulus.

	Number of insured individuals	Number of sigmoid volvulus	Incidence	Chi‐square
	*n*		% (95% CI)	*p*‐Value
Total	327,693	52	0.0159 (0.0119–0.0208)	
Age (years)				0.019
40–69	107,098	12	0.011	
70–79	116,637	13	0.011	
80–89	81,873	22	0.027	
90‐	22,085	5	0.023	
(when limited to >60)	(289,547)	(52)	0.018	
Sex				<0.001
Men	131,655	35	0.027	
Women	196,038	17	0.008	
Hyperlipidemia				0.064
Yes	94,843	9	0.009	
No	232,850	43	0.018	
Hypertension				0.534
Yes	156,026	27	0.017	
No	171,656	25	0.015	
Diabetes				0.714
Yes	43,453	6	0.014	
No	284,241	46	0.016	
Psychiatric disease				<0.001
Yes	41,575	15	0.036	
No	286,118	37	0.013	
Constipation				<0.001
Yes	58,141	32	0.055	
No	269,552	20	0.007	
Long‐Term Care level				<0.001
Independent (no need for support)	266,001	24	0.009	
Support for Nursing Care level 1	26,555	5	0.019	
Nursing Care level 2 to 3	20,243	17	0.084	
Nursing Care level 4 to 5 (severely dependent)	14,894	6	0.040	

**TABLE 2:** Poisson regression analysis on incidence of sigmoid volvulus.

*N* = 327,693	RR, 95% CI (crude)	aRR, 95% CI (full adjusted)	*p*‐Value
Age (years)			
40–69	1	1	
70–79	0.99 (0.45–2.18)	0.70 (0.31–1.56)	0.384
80–89	2.23 (1.19–4.85)	0.95 (0.42–2.10)	0.890
90‐	2.02 (0.71–5.74)	0.54 (0.17–1.73)	0.302
Sex			
Men	3.07 (1.72–5.47)	3.46 (1.92–6.25)	<0.001
Women	1	1	
Psychiatric disease			
Yes	2.79 (1.53–5.08)	1.56 (0.84–2.91)	0.162
No	1	1	
Constipation			
Yes	7.42 (4.24–12.97)	4.63 (2.50–8.58)	<0.001
No	1	1	
Long‐Term Care level			
Independent (no need for support)	1	1	
Support for Nursing Care level 1	2.09 (0.80‐5.47)	1.66 (0.60‐4.65)	0.330
Nursing Care level 2 to 3	9.31 (5.00‐17.33)	6.54 (2.43‐13.57)	<0.001
Nursing Care level 4 to 5 (severe)	4.47 (1.83–10.93)	2.94 (1.10–7.84)	0.031

Abbreviations: aRR, adjusted risk ratio; CI, confidence interval; RR, risk ratio.

## 3 Results

Table 1 shows the incidence rates of SV according to the basic characteristics. In total, 52 patients were newly diagnosed with SV in 2019, with an annual incidence rate of 0.0159% (15.9/100,000). Older individuals, men, and those with psychiatric disease, constipation, and higher disability levels were more likely to be diagnosed with SV, according to the chi‐square test.

The results of the SV incidence rate from Poisson regression analysis are presented in Table 2. In the crude model, aged 80–89 years (RR, 2.23, 95% CI, 1.19–4.85), men (RR, 3.07, 95% CI, 1.72–5.47), psychiatric disease (RR, 2.79, 95% CI, 1.53–5.08), constipation (RR, 7.42, 95% CI, 4.24–12.97), disability (Nursing Care) levels 2–3 (RR, 9.31, 95% CI, 5.00–17.33), and levels 4–5 (RR, 4.47, 95% CI, 1.83–10.93) were significantly associated with the incidence of SV. In the fully adjusted model, men (aRR, 3.46; 95% CI, 1.92–6.25), constipation (aRR, 4.63; 95% CI, 2.50–8.58), disability levels 2–3 (aRR, 6.54; 95% CI, 2.43–13.57), and levels 4–5 (aRR, 2.94; 95% CI, 1.10–7.84) were significantly associated with SV incidence.

**FIGURE 2:**
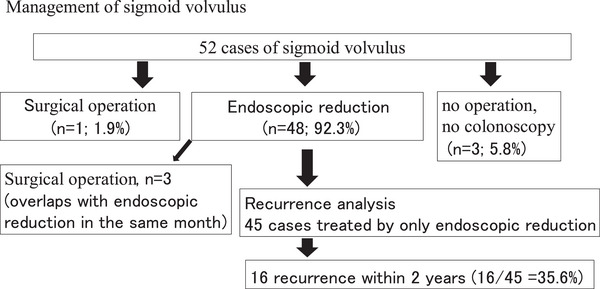
Management of sigmoid volvulus. Of the 52 patients, 48 (92.3%) underwent endoscopic reduction, and three (relatively older patients) did not undergo further treatment. Three patients underwent endoscopic reduction and surgery within the same month. Forty‐five patients who only underwent endoscopic reduction were followed up for 2 years for recurrence analysis. Sixteen patients (35.6%) relapsed.

**FIGURE 3:**
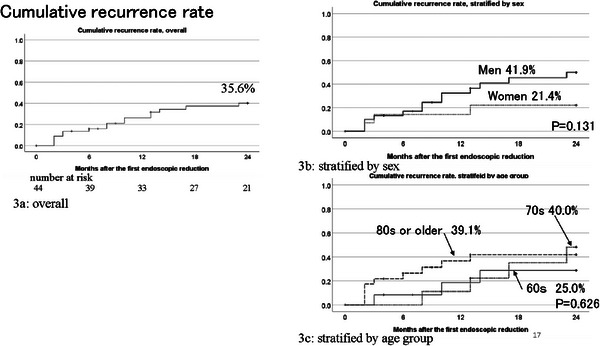
Cumulative recurrence rate overall (a), stratified by sex (b), and age group (c). The results of the log‐rank test are shown in Figures 3b and 3c. Sixteen patients (35.6%) relapsed after the first endoscopic reduction in total (a). Women (21.4%) and patients aged <70 years (25.0%) were less likely to experience relapse after the first endoscopic reduction, though not statistically significant (b and c).

The clinical management of these 52 patients is shown in Figure 2. Of these, 48 patients (92.3%) underwent endoscopic reduction, and three patients (relatively older patients) did not undergo further treatment. Three patients underwent endoscopic reduction and surgery in the same month. Forty‐five patients who underwent endoscopic reduction alone were followed for 2 years for recurrence analysis. Sixteen patients (35.6%) relapsed after the first endoscopic reduction. The results of the Kaplan–Meier analysis stratified by age and sex are shown in Figure 3a–c. Although not statistically significant, women (21.4%) and patients aged < 70 years (25.0%) were less likely to experience relapse after the first endoscopic reduction.

## 4 Discussion

To the best of our knowledge, this is the first study to report the incidence, risk factors, and recurrence rates of SV in Japan using large‐scale community‐based health insurance claims data. Among approximately 330,000 people aged **≥**40 years, the annual incidence rate was 15.9 (per 100,000) in total and 18.0 in those aged >60 years. Considering that patients with SV are transferred to high‐level hospitals that are independently equipped with emergency and GI departments, SV can be common among GI specialists as well as general physicians working in large‐scale hospitals. Therefore, our findings on SV epidemiology may become a useful benchmark, especially in Japan's super‐aging society.

The incidence rate of SV in our study was more than double that of the previous report from Ballantyne et al. [4]. They found that the incidence in Minnesota, USA, between 1960 and 1980 was 1.47 per 100,000 in the total population and 8.76 per 100,000 among those aged **>**60 years. There are several factors that could account for the discrepancy from our results. First, the demographic profile of our study population differs substantially from that in the USA in 1980. According to the US national census, the older population (**≥** 65 years) in 1980 accounted for only 11.3% [14], which was a third of the proportion in our study (32.0%) [12]. Older age is a well‐known risk factor for SV. Second, the universal health insurance system in Japan may have facilitated better access to hospital visits, as patients bear only a small financial burden. Third, medical technology has advanced dramatically since the 1980s. For example, numerous hospitals and clinics in Japan currently have computed tomography (CT) available. In 2017, Japan had 112 CT scanners per 1 million population, four times the average of the Organisation for Economic Co‐operation and Development (OECD) countries [15]. As CT scans are beneficial for identifying SV [16], these factors could explain the discrepancy in SV incidence.

In our community‐based health insurance claims study, SV incidence was significantly associated with men, constipation, and high grades of disability in the multivariable analysis. Our results align with the previous SV risk factors in a hospital‐based study [2, 5, 17, 18]. Lower incidence in women was explained by a capacious pelvis with lax abdominal musculature that can allow the untwisting of a floppy sigmoid [5]. Institutionalized patients were known to lead immobilization of intestine [1, 2]. We first conducted a multivariable analysis of SV and found that older adults and individuals with psychiatric diseases were more likely to develop SV in the crude model; however, these associations were not statistically significant in the multivariate model. This may be because factors such as constipation and disability have a stronger influence on SV risk than age or psychiatric conditions. In addition, our study population likely included people with healthier or less severe diseases than those in hospital‐based studies. These differences may have attenuated the associations among older age, psychiatric diseases, and SV incidence.

Among the 52 SV cases in our study, 48 (92.3%) patients underwent endoscopic reduction, and three patients received surgical treatment in the same month. These managements were in line with SV guidelines [1], which recommend urgent endoscopic reduction as the initial treatment because it is effective in 60–95% of patients [6, 7]. Ören et al. showed that among 827 cases in Turkey, flexible sigmoidoscopy had the highest success rate (78.7%) and the lowest mortality (0.5%) and complication (2.4%) rates [6]. Moreover, compared to Western countries, some Asian countries, including Japan and Korea, have excellent colonoscopy accessibility, with low colonoscopy cost and numerous experienced colonoscopists [19]. Therefore, in the absence of shock or peritonitis on admission, endoscopic reduction should be the first‐line treatment in Japan.

During the 2‐year follow‐up period after the first endoscopic reduction, 16 (35.6%) patients experienced recurrence. Previous studies based on hospital data have reported that the recurrence rate of SV after the first endoscopic reduction ranged from 46.2% to 65.0% [18, 20, 21, 22], which is higher than that found in our results. A study using Medicare claims data in the USA showed that the recurrence rate of SV within 1 year was 36.1% [7], similar to ours. A plausible explanation for the lower recurrence rate in the insurance claims data may be the characteristics of the study population. Compared to a community‐based study, a hospital‐based study might include patients who are, to some extent, more difficult to treat or more likely to experience recurrence. Regarding SV management, previous reports from the UK and New Zealand compared recurrence and survival rates between operative and non‐operative treatments, and recommended early elective surgery for patients without prohibitive comorbidities [23, 24]. However, in Western countries, limited colonoscopy resources exist [19]. According to an OECD report, in many Western countries in the late 2010s, the longest waiting time to get a colonoscopy ranged from 82 to 144 days [25], which contrasts sharply with the higher accessibility in Japan. Therefore, endoscopic reduction can be considered an acceptable approach for SV management in Japan, unless patients present with shock or peritonitis.

A key strength of our study is the use of community‐based, large‐scale health insurance claims data from approximately 330,000 individuals in Toyama, Japan, allowing the investigation of the rare disease SV. Therefore, our study has high generalizability, particularly regarding incidence rates. However, this study has some limitations. First, the diagnosis of SV in our study relies on the validity of coded diagnoses. The health insurance claims data did not contain detailed clinical data, such as defecation status, endoscopic reports, CT findings, or the date of medical treatment. Moreover, claims data may contain an unconfirmed (or ruled out) diagnosis, leading to an overestimation of the incidence rate. To increase diagnostic validity, we assessed 52 patients with SV defined by the diagnostic name and ascertained that 47 patients had both the diagnosis of SV and the billing code for endoscopic reduction (one patient was diagnosed with SV and the billing code for colonoscopy). We assumed that the overestimation of the SV diagnosis hardly occurred in our study. Second, three patients underwent endoscopic reduction and surgery in the same month (Figure 2). These patients were excluded from the recurrence analysis due to uncertainty in the sequence of events. Based on the claims data, we were unable to determine whether these patients initially underwent endoscopic reduction followed by recurrence, or were observed by colonoscopy for necrosis or gangrene of the colon and immediately transferred to the operating room. Because these cases could affect the recurrence rate, future studies using insurance claims data should incorporate detailed clinical information. Third, our study included only the Toyama Prefecture population. Therefore, our findings may not be representative of the entire population of Japan. Moreover, the small number of patients in the recurrence analysis may have resulted in a loss of statistical power. Using nationwide claims data, which includes more patients with SV, may reveal other risk factors for recurrence. Finally, our insurance claims data did not include information on patient death or relocation to other prefectures, which may also affect the recurrence rate. These factors should also be considered in future studies.

Despite these limitations, our community‐based large‐scale study demonstrated the incidence, risk factors, and recurrence rates of SV in Japan. SV is not rare and is commonly seen in large‐scale hospitals, highlighting that the epidemiological data from our study are useful for GI specialists as well as general physicians in managing SV patients. Moreover, owing to the relatively low recurrence rate after endoscopic reduction and the excellent accessibility of colonoscopy, we propose that endoscopic reduction should be the first‐line treatment for patients with SV in Japan.

## Author Contributions

**Masaaki Yamada**: conceptualized and designed the study project and contributed to the statistical analysis of the data. **Masaaki Yamada**, **Michikazu Sekine**, **Haruka Fujinami**, and **Yuchi Motofuji**: contributed to the interpretation of the results. **Haruka Fujinami**, **Yuchi Motofuji**, and **Eiji Shinno**: gave an expertized experience and insight on sigmoid volvulus. **Masaaki Yamada**: composed the manuscript. All authors revised it critically for important clinical contents and approved the final manuscript.

## Ethics Statement

This study was conducted in accordance with the principles of the Declaration of Helsinki. Personal data such as name and birth date were emitted before we received them from Toyama Prefecture. This study was approved by the Institutional Ethical Board of the University of Toyama (R2020178).

## Consent

Not applicable.

## Conflicts of Interest

The authors declare no conflicts of interest.

## Clinical Trial Registration

N/A.

## Supporting information




**TABLE S1** Code of ICD‐10 and drug classification and medical billing code for medical practice in Japan.

## Data Availability

The National Health Insurance Claim “*Kokuho*” and the Advanced Elderly Medical Service System “*Kouki Koureisha Iryo Seido*” data, maintained by the Toyama Prefecture, cannot be shared with third parties. Therefore, we can only provide aggregated data on reasonable requests.
