# Supplementary material for: Epidemiology and Recurrence of Sigmoid Volvulus: Analysis of Health Insurance Claims Data in Japan
Source: DEN Open. 2025 Oct 14;6(1):e70212. doi: 10.1002/deo2.70212 (PMC12520136; doi:10.1002/deo2.70212)
Supplement: Supplementary file 1 — TABLE S1 Code of ICD‐10 and drug classification and medical billing code for medical practice in Japan. [file DEO2-6-e70212-s001.docx]

**Supporting information**

Supporting Table 1

Code of ICD-10 and drug classification and medical billing code for medical practice in Japan.

|  | Diagnosis  in ICD-10 | Drug classification | Medical billing code for medical practice in Japan |
| --- | --- | --- | --- |
| Sigmoid volvulus | K562  (ID20050371) |  |  |
| Colonoscopy |  |  | D313 |
| Endoscopic reduction |  |  | J034-03 |
| Surgical operation in abdomen |  |  | K714, K719, K726, K735 |
| Comorbidity (prescription) |  |  |  |
| hypertension |  | 214 |  |
| hyperlipidemia |  | 218 |  |
| diabetes |  | 396 |  |
| constipation |  | 235 |  |
| psychiatric diseases |  | 117 |  |
| ICD: International Classification of Diseases | | |  |
